# PIM kinase isoform specific regulation of MIG6 expression and EGFR signaling in prostate cancer cells

**DOI:** 10.18632/oncotarget.386

**Published:** 2011-12-21

**Authors:** Allan Siu, Carl Virtanen, Jan Jongstra

**Affiliations:** ^1^ Genetics and Development Division, Toronto Western Research Institute, University Health Network, Toronto, Canada and Department of Immunology, University of Toronto, Toronto, Canada; ^2^ Microarray Centre, University Health Network, Toronto, Canada.

**Keywords:** M-110, PIM-1, MIG6, EGFR, Gefitinib, prostate cancer

## Abstract

The PIM family of oncogenic serine/threonine kinases regulates tumour cell proliferation. To identify proliferative signaling pathways that are regulated by PIM kinases we analyzed gene expression differences in DU-145 and PC3 prostate cancer derived cells induced by treatment with the recently developed highly selective PIM kinase inhibitor M-110. This identified 97 genes the expression of which is affected by M-110 in both cell lines. We then focused on the M-110 induced up regulation of the *MIG6* gene that encodes a negative regulator of EGFR signaling. Here we show that M-110 and the structurally unrelated PIM kinase inhibitor SGI-1776 up regulate *MIG6* in DU-145 and PC3 cells. Knockdown of PIM-1 but not of PIM-2 or PIM-3 also up regulates *MIG6* expression, which identifies *MIG6* as a PIM-1 regulated gene. In agreement with the role of MIG6 protein as a negative regulator of EGFR signaling we found that M-110 treatment inhibits EGF induced EGFR activation and the activation of the downstream ERK MAPkinase pathway. The biological significance of these findings are demonstrated by the fact that co-treatment of DU-145 or PC3 cells with the EGFR tyrosine kinase inhibitor Gefitinib and M-110 or SGI-1776 has synergistic inhibitory effects on cell proliferation. These experiments define a novel biological function of PIM-1 as a co-regulator of EGFR signaling and suggest that PIM inhibitors may be used in combination therapies to increase the efficacy of EGFR tyrosine kinase inhibitors.

## INTRODUCTION

The PIM family of oncogenic serine/threonine kinases consists of three members, PIM-1, PIM-2 and PIM-3. The *pim-1* proto-oncogene was first identified as a locus frequently activated by proviral integration in Moloney murine leukemia virus induced mouse T-cell lymphomas and *pim-2* was identified as a gene frequently activated in secondary transplants of virus induced lymphomas. Pim-3 was identified as a Pim-1 and Pim-2 related kinase. The oncogenic nature of Pim-1 and Pim-2 was confirmed by the observation that transgenic mice over expressing these kinases in the lymphoid system developed lymphomas. Simultaneous over expression of c-myc further increased the frequency of lymphomagenesis [1]. PIM kinases are also involved in the development of solid tumors. PIM-1 and PIM-2 are implicated in prostate cancer development [2, 3], PIM-1 is over expressed in head and neck squamous cell carcinoma and bladder cancer [4, 5] and PIM-3 is over expressed in colorectal, pancreatic and hepatocellular carcinoma [6-8]. PIM-1 and PIM-2 over expression in prostate cancer correlates with tumour progression [2] and over expression of exogenous PIM-1 or PIM-2 in prostate cancer cell lines increases cell proliferation [9, 10]. The molecular mechanisms by which PIM kinases regulate tumour cell proliferation may include the phosphorylation and inactivation of cell cycle inhibitors p27^Kip1^ [10] or p21^cip1^ [11] or the activation of molecules that positively regulate cell cycle progression such as CDC25A, CDC25C or the kinase C-TAK1[12]. PIM kinases may regulate cell viability by phosphorylating the apoptotic proteins BAD and ASK1 [13, 14] and are involved in the regulation of drug resistance [15].

In addition to the identification of individual PIM substrates, the major proliferative signaling pathways that are regulated by PIM kinases are beginning to be identified. We have recently characterized a novel small molecule designated M-110, as a highly selective inhibitor of all three PIM kinase isoforms and showed that M-110 inhibits, through inhibition of PIM-3, but not of PIM-1 or of PIM-2, the phosphorylation of STAT3 on tyrosine residue 705 in the prostate cancer derived cell line DU-145 and the pancreatic cancer derived cell line MiaPaCa2 [16]. STAT3 is an oncogenic transcription factor that is activated by phosphorylation on tyrosine residue 705 and the importance of STAT3 signaling in cell proliferation is well documented [17, 18]. STAT3 is activated by stimulation of IL-6 which is an important autocrine/paracrine growth factor for prostate cancers and M-110 was shown to interfere with IL-6 induced activation of STAT3. However, not all prostate cancer cell lines that are sensitive to M-110 treatment express activated STAT3. For instance the proliferation of 22Rv1 and PC3 cells is inhibited by M-110. However, 22Rv1 cells do not express active STAT3 but express active STAT5 that is not affected by M-110 treatment [16]. PC3 cells do not express STAT3 because of a genomic deletion containing the STAT3 gene [19]. Therefore it is likely that the M-110 induced inhibition of cell proliferation is mediated through inhibition of multiple proliferative pathways in a cell type dependent manner.

EGFR over expression or mutations leads to abnormal EGFR signaling which is linked to the development of many tumours [20]. For instance EGFR expression is increased in a significant proportion of prostate cancer patients and increased expression correlates with increased risk of relapse and progression to castration resistant disease [21-23]. Binding of EGF to the EGFR (ErbB1) results in homodimerization or heterodimerization of the EGFR with any of three EGFR related receptors ErbB2-4. Dimerization leads to phosphorylation of a number of tyrosine residues present in the cytoplasmic portion of the EGFR by the intracellular receptor tyrosine kinase domain. Intracellular proteins with SH2 or phosphotyrosine binding motifs are then recruited to the activated tyrosine phosphorylated receptor to activate a number of proliferative signaling pathways such as the ERK MAPkinase and the PI3-kinase/AKT pathways. Signaling through the EGFR is limited by a number of negative feedback inhibitory proteins that are induced by EGF signaling [24]. One such protein is MIG6 (also known as RALT or ERRFI1) that binds preferentially to the activated EGFR and inhibits its kinase activity [25, 26]. MIG6 may also regulate the internalization and degradation of the EGFR [27, 28]. Here we report that treatment of prostate cancer cells with M-110 or a structurally unrelated PIM kinase inhibitor SGI-1776 increases the expression of *MIG6* RNA and protein and inhibits EGF induced activation of the EGFR and the downstream ERK MAPkinase pathway. Knockdown of PIM-1 but not of PIM-2 or of PIM-3 up regulates *MIG6* expression. This identifies a novel biological function of PIM-1 as a positive co-regulator of the EGF/EGFR/ERK MAPkinase pathway. Furthermore we show synergistic effects of the EGFR tyrosine kinase inhibitor Gefitinib and PIM kinase inhibitors on prostate cancer cell proliferation. These results provide new insights into the oncogenic action of PIM kinases and support the development of PIM isoform specific inhibitors as anti-cancer agents to increase the efficacy of EGFR targeted chemotherapy.

## RESULTS

### PIM-1 regulates expression of the ERBB inhibitor MIG6

**Figure 1:**
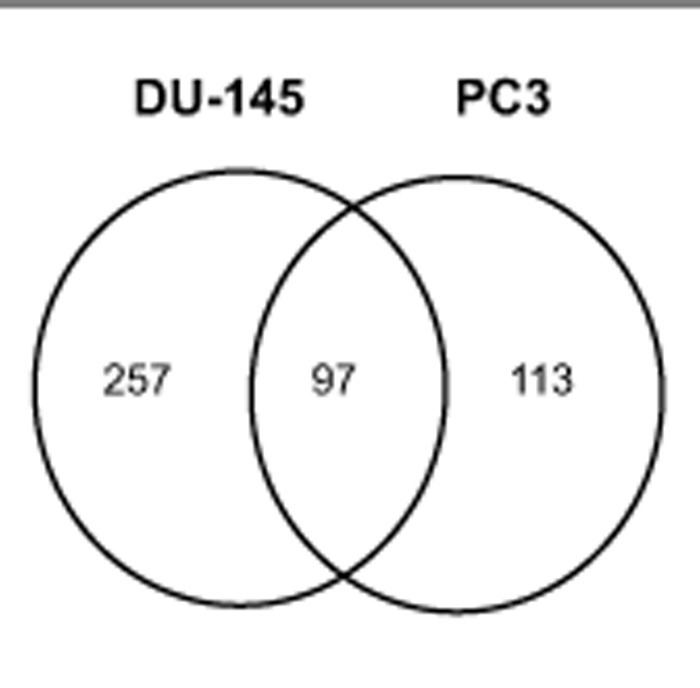
Gene expression differences induced by M-110 treatment of prostate cancer cells A Venn diagram of the number of genes responding to treatment with 10 μM M-110 for 8 hr. Ninety seven gene changes were in common between DU-145 and PC3 cells. Thirty six genes were identified as targets of transcription factors that are regulated by PIM kinases (table 1).

To identify signaling pathways that are affected by M-110 treatment of prostate cancer cells, we used micro array analyses to determine changes in gene expression induced by M-110 treatment of two prostate cancer cell lines, DU-145 and PC3. Cells were treated with 10 μM M-110 or with vehicle only (DMSO) for 8 hrs, and RNA was extracted from 4 independent experiments and analyzed for genome wide changes in gene expression using Agilent 28004 Whole Human Genome expression microarrays. Analyses showed that the expression of 257 probes representing genes changed > 2-fold after treatment of DU-145 cells with M-110 compared to treatment with DMSO only. In PC3 treated cells, 113 gene expression differences were identified and 97 gene expression differences were common among DU-145 and PC3 cells Of these, 59 were up regulated and 38 were down regulated by M-110 treatment in both cell lines (Figure 1 and Supplementary Table 1). RT-qPCR assays for expression of genes included in the 97 common gene set validated the microarray results for 8 genes analyzed (Supplementary Figure 1), showing that the microarray results are of high quality and can be used to predict M-110 induced gene expression differences in DU-145 and PC3 cells with high confidence.

**Figure 2:**
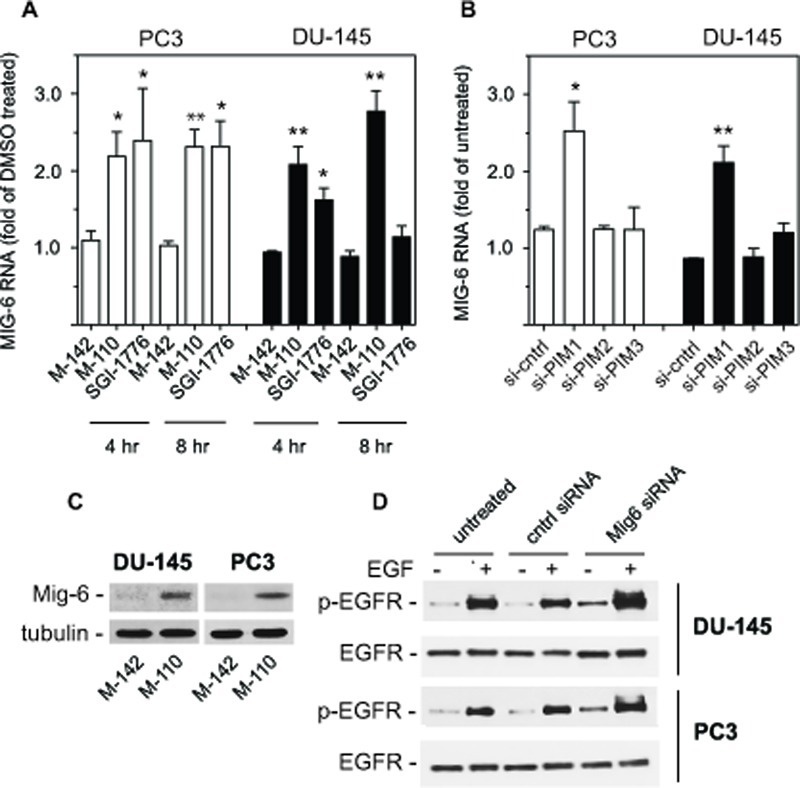
Regulation of *MIG6* gene expression (A) PC3 or DU-145 cells were treated for 4 hr or 8 hr with 10 μM of the indicated compounds and analyzed for *MIG6* RNA by RT-qPCR. (B) Expression of *MIG6* RNA was analyzed by RT-qPCR 40 hr after transfection of PC3 or DU-145 cells with PIM isoform specific siRNAs or a control siRNA. (C) DU-145 or PC3 cells were treated with M-110 or M-142 (10 μM, 16 hr) and whole cell extracts were analyzed for expression of MIG6 protein. Tubulin was used as a loading control. (D) DU-145 or PC3 cells were left untreated or transfected with control or MIG6 siRNA. After treatment with 10 ng/ml EGF for 15 min whole cell lysates were analyzed for EGFR activation as determined by the expression of EGFR phosphorylated on tyrosine residue 1068.

M-110 is a selective inhibitor of PIM kinases and therefore we expected that a significant fraction of gene expression differences would involve genes that are targets of transcription factors that are modulated by PIM kinases. An extensive literature review suggest that this is the case because 36 genes in the 97 common gene set (~ 40 %) affected by M-110 treatment have previously been identified as targets for the PIM modulated transcription factors c-myc [29, 30], c-myc and PIM-1 [31], FOXO3a [32], c-Myb [33, 34] or RUNX1-3 [35, 36] (Table 1). Several genes in the common 36 gene set may contribute to M-110 mediated inhibition of cell proliferation, but we focused on the expression of *MIG6*, because it encodes an important inhibitor of EGFR signaling [37, 38], suggesting that M-110 treatment of prostate cancer cells negatively affects signaling through the EGFR. Figure 2A shows that M-110 treatment for 8 hrs up regulates *MIG6* RNA in PC3 and DU-145 cells, validating the micro array results obtained after 8 hr of treatment. We also included a 4 hr treatment which showed that the effect of M-110 treatment on the expression of *MIG6* is a relatively early event. Treatment with M-142, an inactive derivative of M-110 that does not inhibit PIM kinase or cell proliferation [16] had no effect on the expression of *MIG6* RNA suggesting that up regulation of *MIG6* RNA is a consequence of M-110 mediated PIM inhibition. To further support the involvement of PIM kinases in the regulation of *MIG6* we also treated DU-145 and PC3 cells with SGI-1776, a well defined PIM kinase inhibitor that is not structurally related to M-110 [39]. As shown in Figure 2A treatment of PC3 cells for 4 hr or 8 hr with 10 μM SGI-1776 up regulates *MIG6* RNA expression to a similar extent as treatment with M-110. Treatment of DU-145 cells with SGI-1776 also up regulates *MIG6* RNA after 4 hr but the response of DU-145 cells to 10 μM SGI-1776 appears more transient than the response to 10 μM M-110. Nevertheless the fact that PC3 cells and DU-145 cells up regulate *MIG6* expression after treatment with two unrelated PIM kinase inhibitors, but not with M-142 strongly suggests a role for PIM kinases in the regulation of *MIG6* expression. To determine whether one or more PIM isoforms are involved in the regulation of *MIG6* gene expression we used PIM isoform specific siRNAs to selectively knockdown PIM-1, PIM-2 or PIM-3. We used these isoform specific PIM siRNAs previously to show that PIM-3 is a positive regulator of STAT3-tyrosine 705 phosphorylation [16]. Figure 2B shows that *MIG6* RNA is up regulated in DU-145 and PC3 cells treated with siPIM-1 but not in cells treated with siPIM-2, siPIM-3, or a control siRNA. These results show that PIM-1 but not PIM-2 or PIM-3 inhibits the expression of *MIG6*. The specificity of the PIM isoform specific siRNAs is shown in Supplementary Figure 2A. The up regulation of PIM-2 and PIM-3 by siPIM1 has been observed before [16] but is unlikely to impact the conclusion that *MIG6* is a PIM-1 regulated gene as the PIM-2 and PIM-3 siRNAs have no effect on MIG6 expression.

**Table 1:** Description of the 36 common gene set

Transcr. Factor	Gene	Effect Of M-110 Treatment				
	Symbol	Du-145	Pc3		Gene Name	Access Number
**c-MYC**	ARG2	4.0	2.6	up	arginase, type II	NM_001172
	ATF3	9.9	3.3	up	activating transcription factor 3	NM_001040619
	CCNE2	2.7	4.2	up	cyclin E2	NM_057749
	CXCR4	4.1	2.7	up	chemokine (C-X-C motif) receptor 4	NM_001008540
	DDIT4	4.8	2.6	up	DNA-damage-inducible transcript 4	NM_019058
	FOS	14.2	3.7	up	FBJ murine osteosarcoma viral oncogene homolog	NM_005252
	NDRG1	6.8	7.7	up	N-myc downstream regulated 1	NM_006096
	PPP1R3B	3.0	2.2	up	protein phosphatase 1, regulatory (inhibitor) sub 3B	NM_024607
	TNFAIP3	6.5	5.9	up	tumor necrosis factor, alpha-induced protein 3	NM_006290
	RASFF2	4.6	7.7	up	Ras associated (RalGDS/AF-6) domain family	NM_014737
	SOCS3	3.1	2.3	up	Suppressor of cytokine signaling 3	NM_003955
	CCNB1	3.7	2.3	down	cyclin B1	NM_031966
	CDCA8	2.0	2.5	down	cell division cycle associated 8	NM_018101
	CENPA	4.8	2.6	down	centromere protein A	NM_001809
	HIST1H3B	4.3	3.4	down	histone cluster 1, H3b	NM_003537
	NDC80	2.2	2.9	down	NDC80 homolog	NM_006101
	NUDT6	2.4	2.5	down	nudix	NM_198041
	PLK1	7.5	2.9	down	polo-like kinase 1 (Drosophila)	NM_005030
**c-MYC/PIM-1**	ADM	13.9	5.3	up	adrenomedullin	NM_001124
	BTN2A1	8.6	6.5	up	butyrophilin, subfamily 2, member A1	NM_078476
	FOSB	19.1	2.9	up	FBJ murine osteosarcoma viral oncogene homolog B	NM_006732
	GADD45B	2.7	2.8	up	growth arrest and DNA-damage-inducible, beta	NM_015675
	STARD4	2.1	2.8	up	StAR-related lipid transfer (START) domain containing 4	NM_139164
**FOXO3a**	DUSP1	3.3	7.0	up	dual specificity phosphatase 1	NM_004417
	GADD45A	3.8	2.3	up	growth arrest and DNA-damage-inducible a	NM_001924
	MXI1	5.1	4.2	up	MAX interactor 1	NM_130439
	AURKA	3.2	2.4	down	aurora kinase A	NM_198433
	NEK2	2.2	2.1	down	NIMA related kinase 2	NM_002497
	H1F0	2	2.7	down	Histone H1	NM_005318
**c-myb**	CCNB1	3.9	3.0	down	cyclin B1	NM_031966
	FGF2	2.3	3.0	down	Fibroblast Growth factor-2	NM_002006
	TOP2a	3.6	2.2	down	topoisomarase2	NM_001067
**RUNX**	MIG6	2.2	3.0	up	ERBB receptor feedback inhibitor 1	NM_018948
	ANGPTL4	8.3	26.8	up	angiopoietin-like 4	NM_139314
	ENO2	4.3	2.2	up	enolase 2 (gamma, neuronal)	NM_001975
	CYP1B1	2.1	7.0	down	P450, family 1, subfamily B, polypeptide 1	NM_000104

### M-110 inhibits EGFR activation

**Figure 3:**
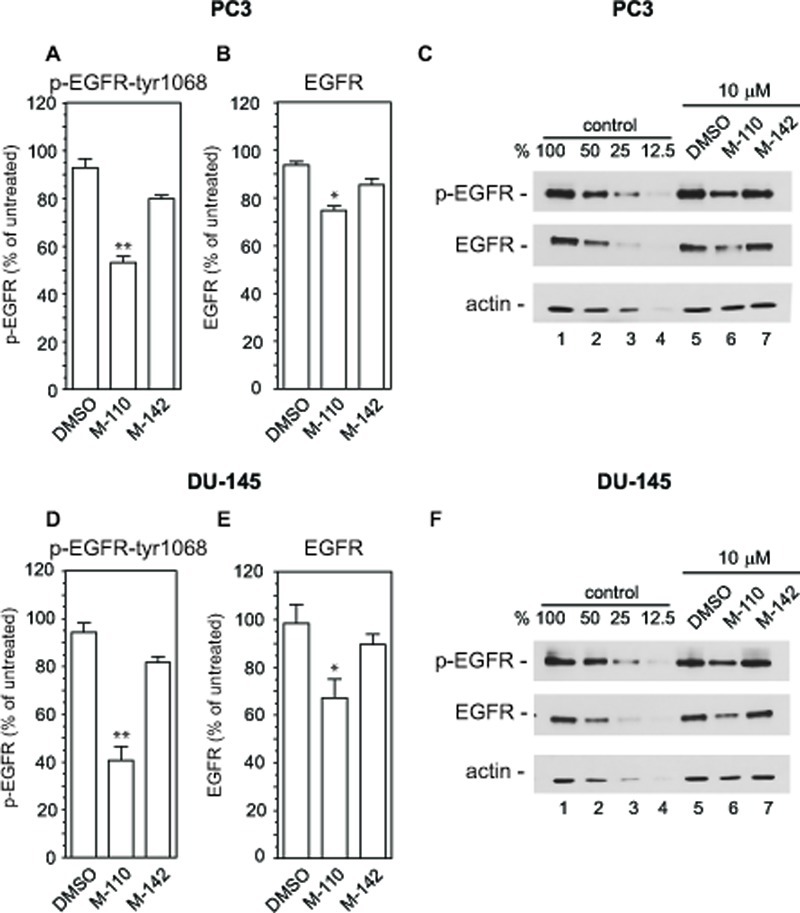
M-110 down regulates EGFRtyr1068 expression PC3 cells (A-C) or DU-145 cells (D-F) were treated with DMSO or with M-110 or M-142 (10 μM, 16 hr) followed by treatment with EGF (10 ng/ml, 15 min) and analyzed for expression of p-EGFRtyr1068 (A,D) or EGFR (B,E). Expression in DMSO or compound + EGF treated cells is expressed as the percentage of the expression in untreated control + EGF treated cells. Results are the average of 3 independent experiments. (C,F) To quantitate the expression of p-EGFRtyr1068, EGFR or actin equal amounts of EGF treated control samples and EGF+compound treated samples were analyzed in lanes 1 and 5-7. Lanes 2-4 contain two-fold serial dilutions of the control sample analyzed in lane 1 to provide a standard curve used for calculating the % change in expression of p-EGFRtyr1068, EGFR and actin in lanes 5-7.

**Figure 4:**
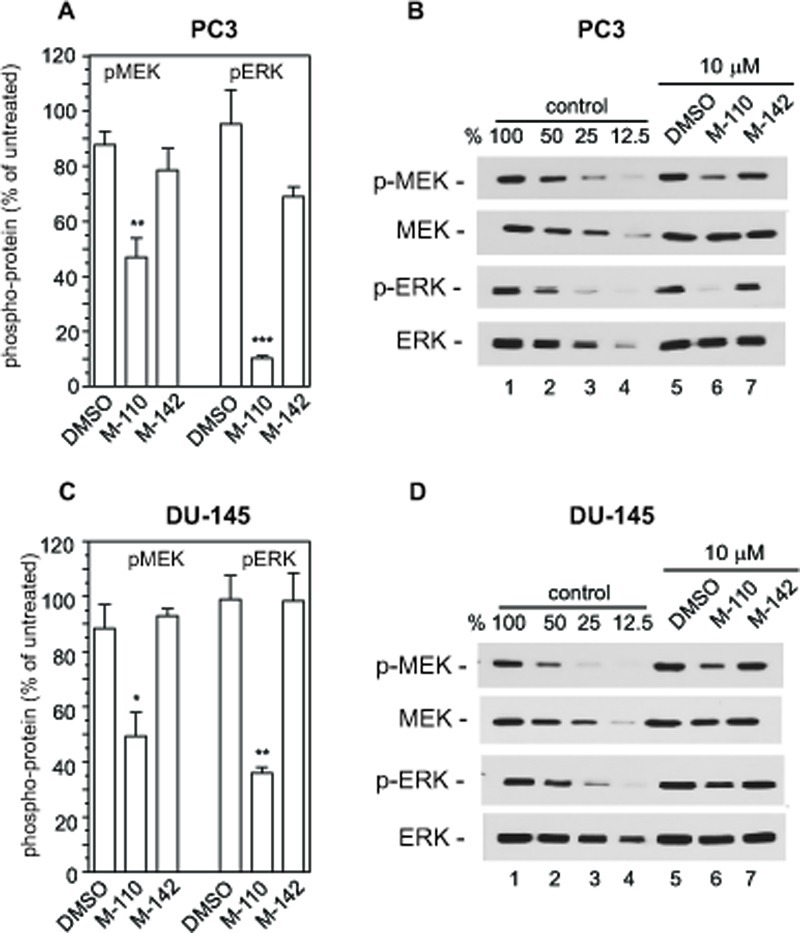
M-110 down regulates EGF induced p-MEK1/2 and p-ERK1/2 PC3 cells (A,B) or DU-145 cells (C,D) were treated with DMSO or with M-110 or M-142 (10 μM, 16 hr) followed by treatment with EGF (10 ng/ml, 15 min) and analyzed for expression of p-MEK1/2 or p-ERK1/2. Expression in DMSO or compound + EGF treated cells is expressed as the percentage of the expression in untreated control + EGF treated cells. Results are the average of 3 independent experiments. (B,D) Quantitation of pMEK1/2, MEK1/2, pERK1/2 and ERK1/2 by Western blotting was as described in the legend to figure 3.

The up regulation of *MIG6* RNA by M-110 results in a robust increase in MIG6 protein as determined by Western blotting of M-110 and M-142 treated PC3 and DU-145 cells (Figure 2C). The specificity of the anti-MIG6 antibodies was verified by analyzing siMIG6 treated DU-145 cells which identified MIG6 as a ~ 52Kd protein (Supplementary Figure 2B). To determine whether the MIG6 protein functions as a negative regulator of EGFR activation, we down regulated expression of *MIG6* RNA and tested whether this resulted in increased EGFR activation as measured by the expression of p-EGFR^tyr1068^. Phosphorylation of tyrosine residue 1068 correlates well with EGFR activation [40]. Figure 2D shows that treatment of DU-145 or PC3 cells with *MIG6* siRNA but not with control siRNA resulted in increased response to EGF, which shows that the MIG6 protein functions as a negative regulator of EGFR signaling in prostate cancer derived cells. Therefore the M-110 induced up regulation of MIG6 is expected to inhibit EGFR activation in PC3 and DU-145 cells. Supplemental Figure 3A,B shows that both cell lines respond robustly to EGF stimulation. To determine whether M-110 treatment leads to inhibition of EGFR activation we pre-treated PC3 or DU-145 cells with M-110 for 16 hrs followed by 15 min of EGF stimulation. We then measured both the changes in tyrosine phosphorylated EGFR as well as possible changes in the expression of total EGFR protein because of the potential for MIG6 to increase EGFR internalization and degradation. Treatment with M-110 before addition of EGF significantly inhibits the EGF induced increase in EGFR^tyr1068^ in PC3 as compared to pre treatment with DMSO, the vehicle for the compounds used (Figure 3A,C) or DU-145 cells (Figure 3D,F). Pre-treatment of PC3 and DU-145 cells with M-110 also reduces the expression of total EGFR protein (Figure 3B,C and E,F). Since the decrease in p-EGFR^tyr1068^ is greater than the decrease in total EGFR (*P<0.05* for both cell lines) the decrease in activated, tyrosine phosphorylated EGFR in PC3 and DU-145 cells may in part be due to decreased phosphorylation and in part to down regulation of the receptor protein. As expected, pre-treatment with M-142 had no significant effect on subsequent EGF stimulation. To determine the effect of decreased EGFR activation on downstream signaling events, we also measured the effect of M-110 treatment on EGF induced activation of the ERK MAP kinase pathway. EGF treatment of PC3 and DU-145 cells increases the expression of phosphorylated activated MEK1/2 and ERK1/2 (Supplementary Figure 3C-F) and treatment with M-110 but not with DMSO or M-142 significantly inhibits the EGF induced activation of both components of the ERK MAPkinase pathway (Figure 4). Collectively these results show that M-110 inhibits EGF signaling through the EGFR as shown by inhibition of EGF induced EGFR^tyr1068^ expression and of EGF induced ERK MAPkinase pathway activation.

### PIM inhibitors increase the efficacy of the EGFR tyrosine kinase inhibitor Gefitinib

**Figure 5:**
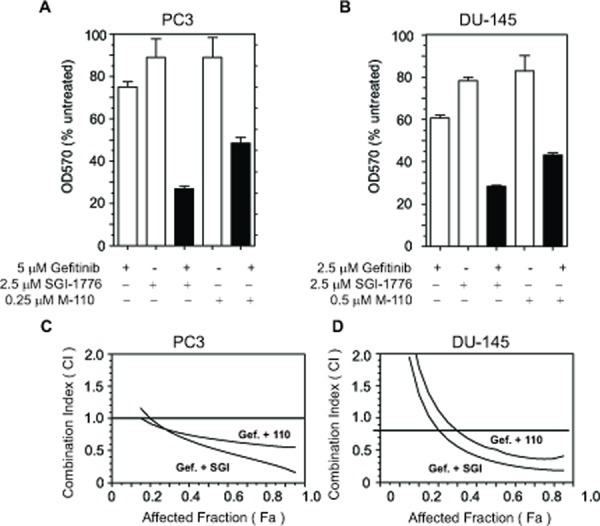
PIM kinase inhibitors increase the efficacy of Gefitinib (A,B) PC3 or DU-145 cells were treated with suboptimal concentrations of Gefitinib, SGI-1776, M-110 or combinations of Gefitinib and SGI-1776 or M-110. Cell proliferation was measured after 72 hrs using the SRB assay. (C,D) Fa-CI plots generated according to Chou and Talalay for PC3 and DU-145 cells treated with gefitinib and SGI-1776 (Gef. + SGI) or Gefitinib and M-110 (Gef. + 110). The CI values are < 1 over a wide range of effects indicating synergistic interactions between Gefitinib and PIM kinase inhibitors.

Both M-110 and SGI-1776 treatment up regulates *MIG6* expression, which results in inhibition of EGFR tyrosine kinase activity. Therefore, our results suggest that the efficacy of EGFR tyrosine kinase inhibitors such as Gefitinib is increased by the addition of M-110 or SGI-1776. To test this hypothesis we first treated PC3 and DU-145 cells with Gefitinib, SGI-1776 or M-110 as single agents or with combinations of Gefitinib and M-110 or Gefitinib and SGI-1776. The concentrations of the compounds used as single agents or in combinations were used at suboptimal concentrations close to 0.5x their respective IC50s for growth inhibition. The IC_50_s are shown in Table 2. Figure 5A,B shows that the effects of the combination treatments are significantly greater than the effects of the single agent treatments suggesting additive or synergistic interactions. In a second set of experiments we then determined whether the interactions between Gefitinib and the two PIM kinase inhibitors are synergistic or additive using the combination index (CI) method of Chou and Talalay [41]. Gefitinib was mixed with SGI-1776 or M-110 at ratios similar to those shown in panels A and B with the highest concentrations at 4x their respective IC50s. PC3 and DU-145 cells were then treated with two-fold serial dilutions of these drug combinations or of the single agents and the CI values were calculated at different effect levels (affected fraction or Fa) using the CompuSyn software developed by Chou and Martin. The CI-Fa plots presented in Figure 5C,D show that the CI values are < 1 over a large range of Fa values indicating synergistic interactions between Gefitinib and SGI-1776 or M-110. It is not clear why the Gefitinib / PIM kinase combinations show only additive or even significant antagonism at low Fa values. However these Fa values have little potential clinical applications and our experiments clearly demonstrate that under conditions where cell proliferation is inhibited by > 80% Gefitinib and PIM kinase inhibitors act synergistically. These experiments illustrate the biological significance of our finding that *MIG6* is a PIM-1 regulated gene and suggests a way to increase the efficacy of EGFR inhibitor based chemotherapy.

**Table 2:** IC50 values compounds for proliferation of PC3 and DU-145 cells

IC50 (μM)	PC3	DU-145
**Gefitinib**	9.6	5.5
**SGI-1776**	4.4	5.8
**M-110**	0.41	0.98

## DISCUSSION

Our results document two important new findings. First, using two structurally unrelated and highly selective small molecule PIM kinase inhibitors and PIM kinase isoform specific siRNAs we show that the regulation of *MIG6* involves PIM-1 but not PIM-2 or PIM-3. Together with the negative effect of M-110 on EGFR activation this defines a novel biological function for PIM-1 as a positive co-regulator of signaling through the EGFR. Second, we show that PIM kinase inhibitors increase the efficacy of the EGFR inhibitor Gefitinib.

The finding that PIM-1 but not PIM-2 or PIM-3 is involved in the regulation of MIG6 expression is significant as little is known about PIM isoform specific functions. Two rare examples of studies that were able to define PIM isoform specific functions showed that PIM-1 but not PIM-2 regulates CXCR4 membrane expression [42] and that PIM-2 but not PIM-1 confers resistance to rapamycin mediated inhibition of IL-3 stimulated bone marrow cell growth [43]. The role of PIM-3 was not specifically addressed in these studies. Using PIM isoform specific siRNAs we have previously shown that PIM-3 but not PIM-1 or PIM-2 is a co-regulator of STAT3 activation in DU-145 cells [16]. Combined with our current data that PIM-1 but not PIM-2 or PIM-3 is co-regulator of EGFR activation in DU-145 and PC3 cells, it is evident that PIM-1 and PIM-3 can independently regulate two important proliferative signaling pathways even when expressed in the same cells. The mechanisms underlying the PIM-1 isoform specific regulation of *MIG6* are unknown. *MIG6* is a RUNX1 regulated gene [36] and PIM-1 is known to phosphorylate RUNX3 in vitro and in HEK293 cells. PIM-1 was also shown to phosphorylate RUNX1 in vitro [44, 45]. The PIM-1 phosphorylation sites on RUNX1 have not been rigorously identified but it is possible that the isoform specificity is determined at the level of RUNX1 phosphorylation. One could speculate that PIM-1 and PIM-3 phosphorylate different residues of RUNX1 that may have different consequences for the activity of RUNX1. If so, the differences are likely to be quantitative rather than qualitative as was shown for the phosphorylation of the BAD protein in which different residues are phosphorylated by PIM-1 and PIM3 with PIM-1 displaying a preference for residue Ser112 and PIM-3 displaying a preference for Ser136 and Ser155 [46]. However it should be noted that RUNX1 does not contain an amino acid sequence with high homology to the Lys/Arg rich PIM consensus phosphorylation site and the phosphorylation of RUNX1 by PIM-1 at an atypical site may be PIM-1 specific.

The *MIG6* gene expression is also up regulated by EGF/EGFR stimulation in an ERK MAPkinase dependent manner [47] an observation that prompted the classification of *MIG6* as a negative feedback inhibitor of EGF/EGFR signaling. To rule out that the EGF induced up regulation of *MIG6* influences our measurements of the effect of M-110 or SGI-1776 on up regulation of MIG6, we determined the time course of EGF induced *MIG6* RNA expression by RT-qPCR in DU-145 and PC3 cells and found that increases in *MIG6* RNA are first detectable between 30 and 60 min after addition of EGF (Supplementary Figure 4). It is thus unlikely that our results on PIM inhibitor mediated inhibition of EGFR activation measured after 15 min of EGF stimulation are influenced by EGF up regulated *MIG6*.

Consistent with the known functions of MIG6, down regulation of *MIG6* by siRNA increased EGF induced activation of EGFR. Signaling through the EGFR is involved in many aspects of tumour development, contributing to cancer cell proliferation, cell survival and cell motility and invasion [20, 48]. For instance, growth of early prostate tumours is androgen dependent and is therefore sensitive to androgen ablation. However upon relapse the disease almost invariably progresses to castrate resistant prostate cancer (CRPC) in which prostate tumours acquire the capacity to grow in castrate levels of hormone. Expression of the EGFR is up regulated in CRPC [22, 23] suggesting that growth of these late stage tumours is at least in part driven by exaggerated signaling through the EGFR. Antibodies against the ligand binding domain of EGFR (Erbitux) or small molecules that inhibit the EGFR tyrosine kinase activity (Gefitinib, Erlotinib) have been developed as therapeutic agents for treatment of EGFR driven tumours and these reagents are successfully employed in the clinic, especially against subsets of patients with NSCLC. However the results of EGFR kinase inhibitors for the treatment of CRPC have been somewhat disappointing [49, 50]. This lack of in vivo efficacy is reflected by the high in vitro IC_50_ values of Gefitinib for growth inhibition of DU-145 and PC3 cells. Thus our results that combinations of Gefitinib and PIM inhibitors show synergistic effects on growth inhibition therefore has potential therapeutic implications for treatment of CRPC patients since the effective dose of Gefitinib in combination with PIM inhibitors can be lowered to 2.5 μM, a concentration that may be attainable in plasma of patients treated with high dose once daily Gefitinib [51]. In addition to the potential use of Gefitinib / PIM kinase inhibitor combination in the treatment of CRPC, this combination may be especially attractive in treatment of EGF driven tumours such as NSCLC in which Gefitinib as a single agent already has significant activity. Adding a PIM inhibitor leading to up regulation of MIG6 may have a broader effect on EGFR signaling than treatment with EGFR specific tyrosine kinase inhibitors alone because MIG6 also inhibits signaling through the EGFR related receptor ERBB2 [52]. Up regulation of ERBB2 is an important factor of the acquired resistance to EGFR targeted therapies. Thus addition of PIM inhibitors not only increases the initial efficacy of EGFR targeted therapies but by inhibiting HER2 may also significantly increase the disease free interval between treatment and relapse.

The identification of PIM-1 as a positive regulator of EGF/EGFR signaling expands our knowledge of oncogenic signaling pathways that are controlled by single PIM kinase isoforms. This suggest that developing PIM isoform specific inhibitors will not only allow a further characterization of PIM isoform specific functions but also allow a targeted approach to cancer treatment through the rational design of combination therapies.

## MATERIALS AND METHODS

### Microarray Analyses

200 ng of starting material for each sample as well as Human Universal Reference RNA were amplified and labeled with Cy5 and Cy3 dyes, respectively, and hybridized to Agilent 28004 Human Whole Genome microarrays according to the standard manufacturers protocols. Microarray slides were scanned on a G2565C DNA Scanner and quantified using Feature Extraction Software version 10.7.1.1.3 (Agilent). Quality of individual samples was assessed in R (v2.10) using the ArrayQualityMetrics Bioconductor package [53]. Data were then imported into GeneSpring (v11.5.1, Agilent) for further analyses.

Normalization consisted of an Agilent Spatial Detrending normalization followed by a median centred “per-gene” normalization. All statistics were carried out on log base 2 converted data. Data were first filtered such that only probes that were in the upper 80^th^ percentile of measured expression in 75 percent of the samples for any 1 out of the 4 categories (PC3 or DU145 M110 treated or DMSO control) were used, leaving 37678 probes out of the 42545 probes found on the array for further analysis. Statistical testing consisted of a 2-way ANOVA (treatment type and cell type) with a Benjamini and Hochberg [54] multiple testing correction False Discovery Rate set to p<0.05. 7988 probes were found to be significantly different between the cell types and 401 were found between the treatment versus control category. Of the 401 probes found different between the M110 treatment and DMSO control, 257 probes in the DU145 cell type and 113 in the PC3 cell type were greater than 2 fold different and 97 probes were found in common between the two cell types.

### Cell culture

DU-145 and PC3 cells were purchased from the American Type Culture Collection (Rockville, MD, USA) and grown in MEM supplemented with 10% FCS. All media were supplemented with 100 units/ml of penicillin, 0.1 mg/ml streptomycin, 2 mM glutamine and 1xMEM non-essential amino acids (Sigma-Aldrich, Oakville, Ontario, Canada).

### Reagents and Antibodies

Compound M-110 was synthesized by Sundia MediTech Company (Shanghai, P.R.China). M-142 was synthesized at the Ontario Institute for Cancer Research (Toronto, ON, Canada). Gefitinib and SGI-1776 were from Selleck Chemicals (Houston, TX, USA). Recombinant human EGF was from Peprotech (Montreal, QC, Canada). Antibodies specific for MIG6, EGFR, pEGFR^tyr1068^, ERK1/2, p-ERK1/2 and p-MEK1/2 were from Cell Signaling Technology (Danvers, MA, USA). Tubulin and MEK1/2 antibodies were from SantaCruz Biotechnologies (Santa Cruz, CA, USA). Anti-actin was from Sigma-Aldrich.

### EGF stimulation

Cells (0.5×10^5^) were plated in 12-well plates and allowed to adhere for 16-20 hr. Cells were then starved in serum free medium for 48 hrs. Compounds were added to the cultures during the last 16 hrs of starvation after which the cells were stimulated for different time intervals with 10 ng/ml EGF. Whole cell extracts were then prepared by lyses in 75 μl 2xLaemmli sample buffer and analyzed by Western blotting. Statistical significance of differences in expression were determined using GraphPad InStat 3.10 (GraphPad Software, Inc., San Diego, CA USA) by one-way ANOVA tests with Dunnett's post tests comparing results from individual compound treated samples to the results of M-142 or DMSO treated controls.

### siRNA transfections

DU-145 or PC3 cells (2.5x10^5^) were transfected in 6-well plates with siRNAs specific for PIM-1, PIM-2, PIM-3, MIG6 or control siRNA (FlexiTube siRNAs Hs_PIM1_6, Hs_PIM2_5, Hs_PIM3_1, Hs_Mig6_4 or Ctrl_AllStars_1 siRNA respectively) from Qiagen Inc. (Mississauga, ON, Canada) using the HiPerFect transfection reagent (Qiagen). RNA expression was analyzed by Quantitative Real-Time PCR 40 hrs after addition of siRNAs as described [16].

### Cell proliferation assay and synergy determinations

Growth inhibition of test compounds on prostate cancer derived cell lines was determined using the Sulphorhodamine B (SRB) assay as described [55] except that 0.1% acetic acid was used for staining cells with SRB and removing unbound SRB. Synergy between Gefitinib and PIM kinase inhibitors was determined according to Chou and Talalay using the CompuSyn software (Chou, T.C. and Martin, N. ComboSyn, Inc. Paramus, NJ, USA. www.combosyn.com)

## Supplementary Figures and Tables

Supplementary Figure 1

Supplementary Figure 2

Supplementary Figure 3

Supplementary Figure 4

Supplementary Table 1
