# Supplementary material for: PIM kinase isoform specific regulation of MIG6 expression and EGFR signaling in prostate cancer cells
Source: Oncotarget. 2011 Dec 21;2(12):1134–44. doi: 10.18632/oncotarget.386 (PMC3282072; doi:10.18632/oncotarget.386)
Supplement: Supplementary Figure 1 [file oncotarget-02-1134-s001.pdf]

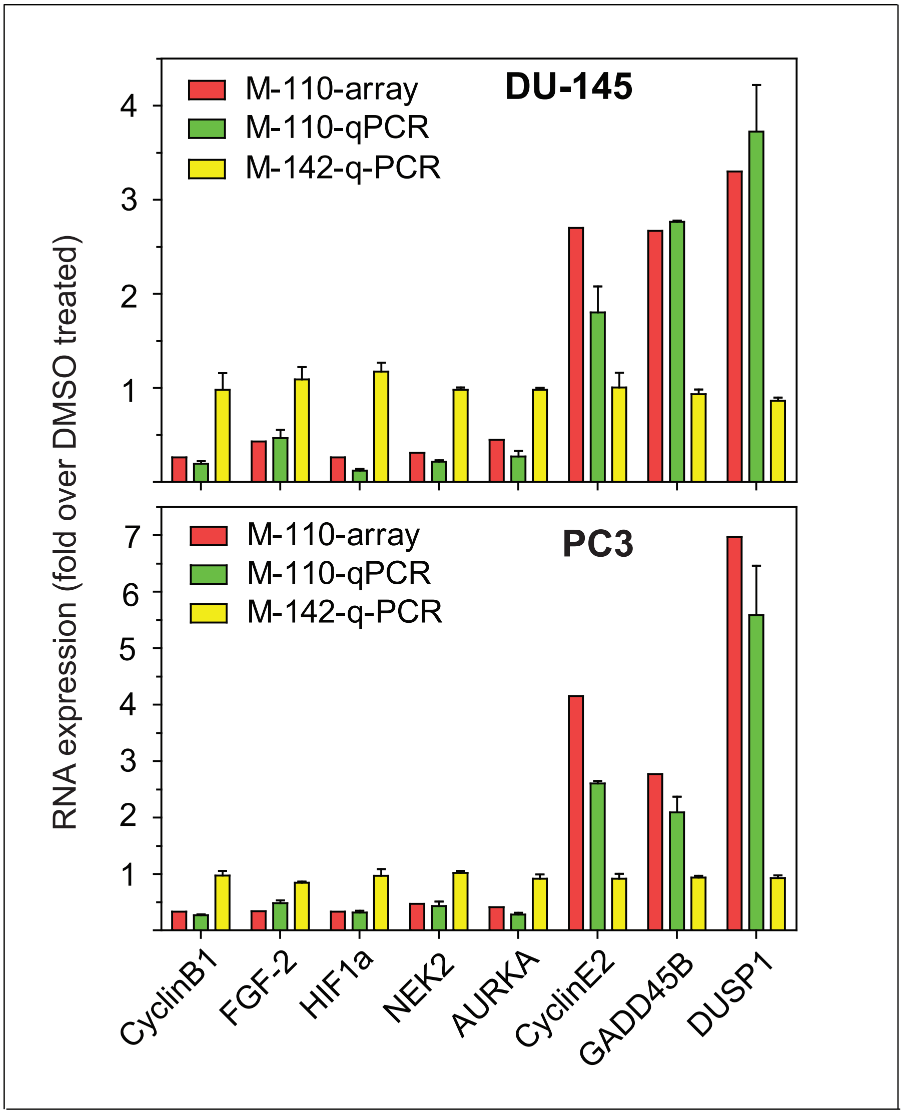

Supplementary Figure 1. Validation of the micro array results for 8 genes contained in the 97 common gene set.Red bars indicate the micro array results, the green and yellow bars are the results of validation experiments using RT-qPCR on RNA from M-110 and M-142 treated cells. M-142 is an inactive compound and serves as a negative control.
