# Supplementary material for: PIM kinase isoform specific regulation of MIG6 expression and EGFR signaling in prostate cancer cells
Source: Oncotarget. 2011 Dec 21;2(12):1134–44. doi: 10.18632/oncotarget.386 (PMC3282072; doi:10.18632/oncotarget.386)
Supplement: Supplementary Figure 2 [file oncotarget-02-1134-s002.pdf]

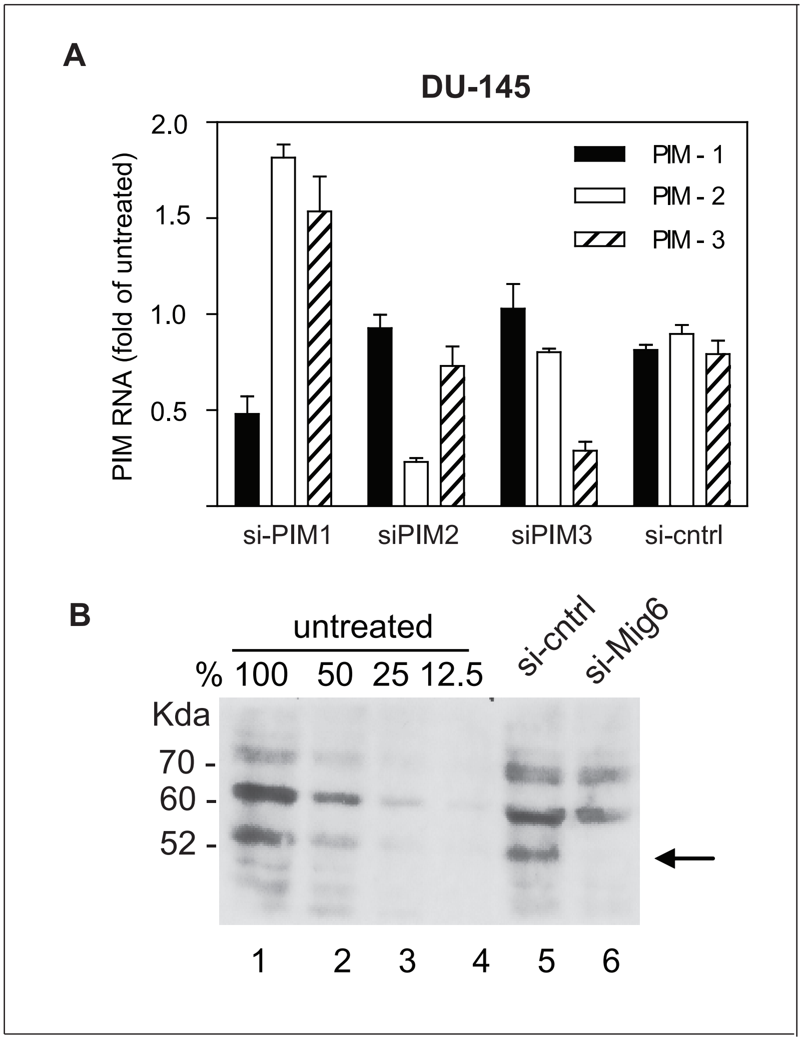

Supplementary Figure 2. (A) Specificity of PIM isoform specific siRNAs. (B). DU-145 cells were treated with MIG6 siRNA or a control siRNA and analyzed by Western blotting. The arrow indicates the MIG6 specific band in lane 6. Lanes 2 and 3 and 4 contain 2-fold, 4-fold and 8-fold dilutions of the untreated sample shown in lane 1. Comparison of lanes 6 and 3 show that the siMig6 transfection reduces expression of MIG6 protein by at least 75 %.
