# Supplementary material for: PIM kinase isoform specific regulation of MIG6 expression and EGFR signaling in prostate cancer cells
Source: Oncotarget. 2011 Dec 21;2(12):1134–44. doi: 10.18632/oncotarget.386 (PMC3282072; doi:10.18632/oncotarget.386)
Supplement: Supplementary Figure 3 [file oncotarget-02-1134-s003.pdf]

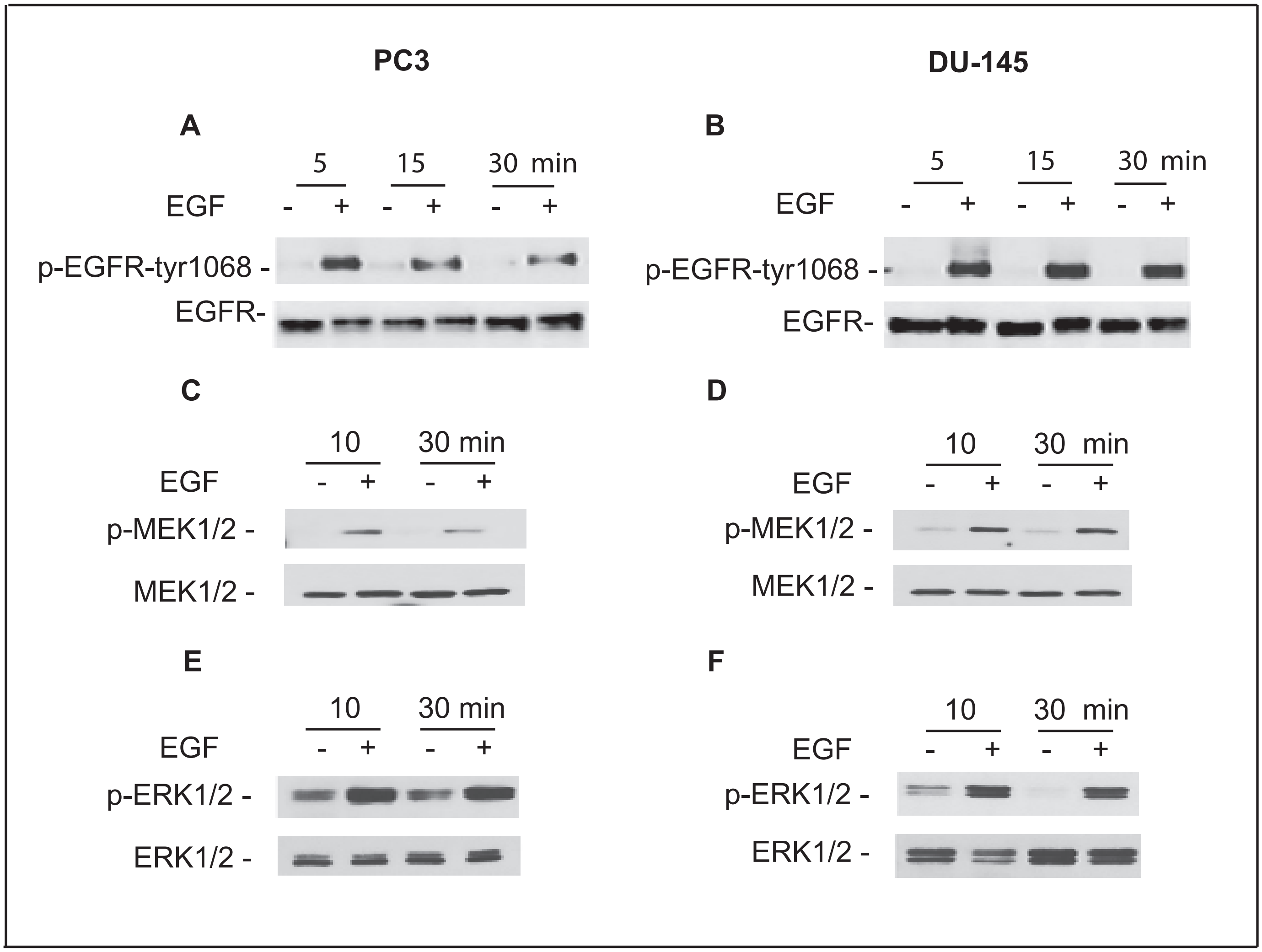

Supplementary Figure 3. PC3 and DU-145 respond to EGF. Cells were treated with 10 ng/ml EGF for the indicated times which resulted in the up regulation of EGFR-tyr1068 (A,B), phospho-MEK1/2 (C,D) and phospho-ERK1/2 (E,F)
