# Supplementary material for: PIM kinase isoform specific regulation of MIG6 expression and EGFR signaling in prostate cancer cells
Source: Oncotarget. 2011 Dec 21;2(12):1134–44. doi: 10.18632/oncotarget.386 (PMC3282072; doi:10.18632/oncotarget.386)
Supplement: Supplementary Figure 4 [file oncotarget-02-1134-s004.pdf]

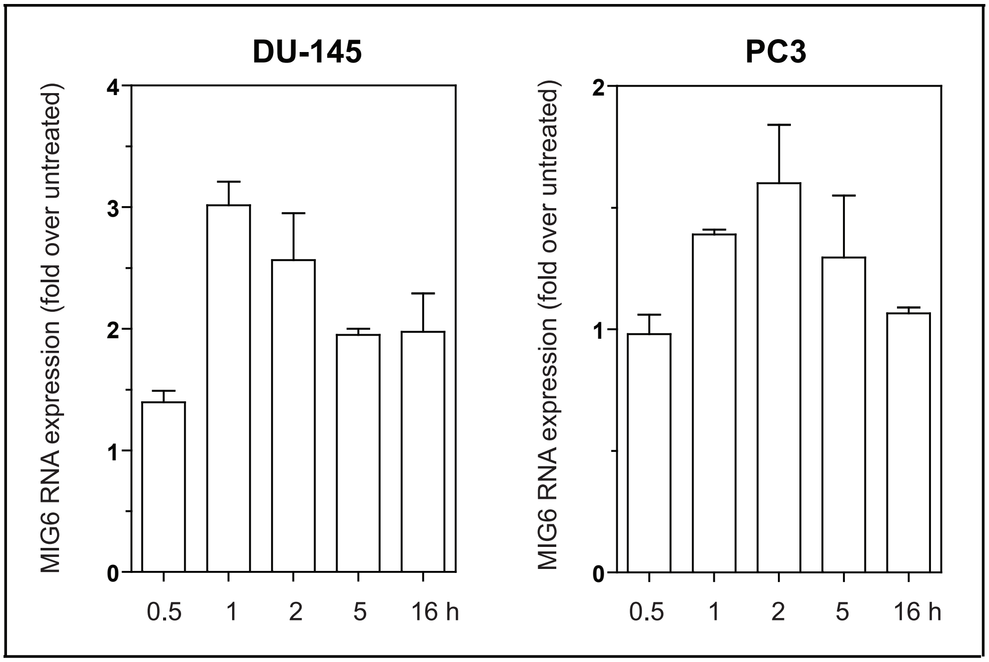

Supplementary Figure 4. EGF dependent up regulation of *MIG6*. DU-145 and PC3 cells were treated with 10 ng/ml EGF for the indicated times. *MIG6* RNA expression was measured by RT-qPCR.
