# Supplementary material for: PIM kinase isoform specific regulation of MIG6 expression and EGFR signaling in prostate cancer cells
Source: Oncotarget. 2011 Dec 21;2(12):1134–44. doi: 10.18632/oncotarget.386 (PMC3282072; doi:10.18632/oncotarget.386)
Supplement: Supplementary Table 1 [file oncotarget-02-1134-s005.pdf]

Supplementary Table 1 The table lists 59 and 38 genes the expression of which are up or down regulat at least 2-fold after treatment of DU-145 and PC3 cells with 10 μM M-110.

Upregulated

		**DU-145**	**PC3**	
		fold	increase	
GeneSymbol	ProbeName			GenbankAccession
AATK	A_23_P10	2.29	2.02	NM_001080395
ADM	A_23_P12	13.86	5.33	NM_001124
ANGPTL4	A_33_P32	8.25	26.83	NM_139314
ANKRD37	A_24_P23	13.92	3.23	NM_181726
ARG2	A_23_P12	3.96	2.63	NM_001172
ARID3A	A_33_P32	2.32	2.41	NM_005224
ATF3	A_23_P34	65.58	4.44	NM_001040619
ATF3	A_33_P32	9.93	3.33	NM_001674
BEND5	A_23_P90	2.06	2.17	NM_024603
BHLHE40	A_24_P26	3.00	2.00	NM_003670
BIK	A_23_P40	2.78	2.12	NM_001197
BTN2A1	A_23_P50	8.58	6.52	NM_078476
C20orf46	A_23_P12	5.78	2.87	NM_018354
C2orf72	A_33_P32	9.07	2.86	NM_001144994
C4orf47	A_33_P32	5.64	2.38	NM_001114357
C7orf68	A_23_P20	7.39	2.60	NM_013332
CCNE2	A_33_P32	2.70	4.15	NM_057749
CHAC1	A_33_P33	4.48	3.37	NM_024111
CSRNP1	A_33_P32	6.41	2.01	NM_033027
CXCR4	A_23_P10	4.06	2.71	NM_001008540
DDIT4	A_23_P10	4.81	2.61	NM_019058
DPYSL4	A_23_P33	20.24	2.90	NM_006426
DUSP1	A_23_P11	3.27	6.96	NM_004417
EGR1	A_23_P21	21.39	5.84	NM_001964
ELF3	A_23_P10	2.31	2.62	NM_004433
ENO2	A_24_P23	4.27	2.23	NM_001975
ERRFI1	A_23_P46	2.18	2.96	NM_018948
FAM115C	A_32_P21	28.51	2.26	NM_173678
FOS	A_23_P10	14.23	3.65	NM_005252
FOSB	A_23_P42	19.15	2.92	NM_006732
FUT11	A_23_P41	3.36	2.26	NM_173540
GADD45A	A_23_P23	3.83	2.31	NM_001924
GADD45B	A_24_P23	2.68	2.77	NM_015675
GPR155	A_32_P13	6.34	2.27	NM_001033045
HK2	A_32_P17	5.03	2.96	NM_000189
IGFBP3	A_23_P21	3.68	3.11	NM_001013398
INSIG2	A_33_P33	3.60	2.18	NM_016133
INSIG2	A_24_P94	2.54	2.09	NM_016133
KCTD11	A_23_P35	5.63	3.37	NM_001002914
KISS1R	A_33_P32	40.57	2.71	NM_032551
LOX	A_23_P12	7.13	2.89	NM_002317
MXD1	A_24_P37	3.03	2.13	NM_002357
MXI1	A_33_P33	5.08	4.19	NM_130439
MXI1	A_23_P16	3.16	2.21	NM_130439
NDRG1	A_23_P20	6.80	7.66	NM_006096
PFKFB4	A_24_P36	8.36	4.25	NM_004567
PPP1R3B	A_24_P20	2.95	2.21	NM_024607
PPP1R3G	A_33_P33	3.60	2.50	NM_001145115
RELT	A_24_P38	3.59	2.16	NM_032871
RIMKLA	A_23_P34	4.43	2.42	NM_173642
RORA	A_23_P26	12.33	2.48	NM_134260
SESN2	A_23_P35	2.09	2.09	NM_031459
SNX33	A_23_P25	2.99	2.16	NM_153271
SOCS3	A_23_P20	3.06	2.26	NM_003955
STARD4	A_23_P32	2.08	2.83	NM_139164
TNFAIP3	A_24_P15	6.48	5.94	NM_006290
ZBTB1	A_23_P99	5.91	2.33	NM_014950
	A_19_P00	2.94	2.51	lincRNA:chr6:22136433-22147296 reverse strand
	A_33_P32	2.86	5.38	perilipin 2 [Source:HGNC Symbol;Acc:248] [ENST00000380464]

Downregulated

		**DU-145**	**PC3**	
		fold	decrease	
GeneSymbol	ProbeName			GenbankAccession
ARHGAP11A	A_24_P29	4.34	3.71	NM_014783
ASPM	A_33_P32	3.72	2.32	NM_018136
ASPM	A_23_P52	2.41	2.41	NM_018136
AURKA	A_23_P13	3.21	2.43	NM_198433
AURKAPS1	A_33_P32	3.12	2.20	NR_001587
CCNB1	A_33_P34	3.87	3.04	NM_031966
CCNB1	A_23_P12	3.66	2.29	NM_031966
CDCA8	A_23_P37	2.00	2.47	NM_018101
CENPA	A_24_P41	4.82	2.64	NM_001809
CENPE	A_23_P25	2.10	2.44	NM_001813
CYP1B1	A_33_P32	2.10	6.98	NM_000104
DEPDC1	A_23_P20	2.17	3.27	NM_017779
FAM72A	A_33_P32	4.37	2.55	NM_001123168
FAM72D	A_32_P15	5.83	2.62	NM_207418
FAM83D	A_23_P32	3.13	2.69	NM_030919
FGF2	A_33_P34	2.25	2.97	NM_002006
FLJ38576	A_33_P32	4.34	2.39	AK095895
G2E3	A_23_P99	3.02	2.46	NM_017769
GRPR	A_24_P35	3.09	2.26	NM_005314
HIF1A	A_24_P56	3.90	3.06	NM_181054
HIST1H3B	A_23_P93	4.31	3.42	NM_003537
HIST1H3E	A_23_P70	2.36	2.21	NM_003532
HIST1H3H	A_33_P32	2.41	3.58	NM_003536
HIST2H2AC	A_23_P30	2.47	3.39	NM_003517
KIF14	A_33_P32	2.19	2.26	NM_014875
KIF18A	A_33_P32	2.82	4.24	NM_031217
KIF20A	A_23_P25	2.82	2.10	NM_005733
KIF23	A_23_P48	2.18	2.08	NM_138555
NDC80	A_23_P50	2.24	2.95	NM_006101
NEK2	A_23_P35	2.24	2.14	NM_002497
NUDT6	A_23_P15	2.40	2.48	NM_198041
PLK1	A_33_P32	7.52	2.88	NM_005030
PLK1	A_23_P11	4.00	2.03	NM_005030
PSRC1	A_23_P46	2.76	2.09	NM_032636
TOP2A	A_23_P11	3.63	2.17	NM_001067
TRIM6	A_24_P38	3.11	2.20	NM_001003818
TTK	A_23_P25	2.08	2.07	NM_003318
	A_19_P00	2.09	2.15	lincRNA:chr7: 7928975-7996750 reverse strand
